# The Global Relationship between Chromatin Physical Topology, Fractal Structure, and Gene Expression

**DOI:** 10.1038/srep41061

**Published:** 2017-01-24

**Authors:** L. M. Almassalha, A. Tiwari, P. T. Ruhoff, Y. Stypula-Cyrus, L. Cherkezyan, H. Matsuda, M. A. Dela Cruz, J. E. Chandler, C. White, C. Maneval, H. Subramanian, I. Szleifer, H. K. Roy, V. Backman

**Affiliations:** 1 Department of Biomedical Engineering, Northwestern University, Evanston, Illinois, 60208, USA; 2 Section of Gastroenterology, Boston Medical Center/Boston University School of Medicine, Boston, Massachusetts, 02118, USA; 3 Department of Biochemistry and Molecular Biology, University of Southern Denmark, Campusvej 55, DK-5230 Odense M, Denmark; 4 Department of Chemistry, Northwestern University, Evanston, Illinois, 60208, USA; 5 Chemistry of Life Processes Institute, Northwestern University, Evanston, Illinois, 60208, USA

## Abstract

Most of what we know about gene transcription comes from the view of cells as molecular machines: focusing on the role of molecular modifications to the proteins carrying out transcriptional reactions at a loci-by-loci basis. This view ignores a critical reality: biological reactions do not happen in an empty space, but in a highly complex, interrelated, and dense nanoenvironment that profoundly influences chemical interactions. We explored the relationship between the physical nanoenvironment of chromatin and gene transcription *in vitro*. We analytically show that changes in the fractal dimension, *D*, of chromatin correspond to simultaneous increases in chromatin accessibility and compaction heterogeneity. Using these predictions, we demonstrate experimentally that nanoscopic changes to chromatin *D* within thirty minutes correlate with concomitant enhancement and suppression of transcription. Further, we show that the increased heterogeneity of physical structure of chromatin due to increase in fractal dimension correlates with increased heterogeneity of gene networks. These findings indicate that the higher order folding of chromatin topology may act as a molecular-pathway independent code regulating global patterns of gene expression. Since physical organization of chromatin is frequently altered in oncogenesis, this work provides evidence pairing molecular function to physical structure for processes frequently altered during tumorigenesis.

Transcription determines the molecular activities and functions of a cell by regulating the abundance and types of proteins available. It has become increasingly accepted that to regulate transcription of a gene, the target sequence of DNA must be accessible within the nucleus to polymerases and transcription factors^1^,^2^,^3^. Despite accessibility being largely influenced by the physical organization of the nuclear nanoarchitecture, most of what we know about key genomic processes, including transcription, comes from viewing cells as molecular machines^4^,^5^,^6^,^7^,^8^. However, molecular interactions do not happen in idealized conditions, but in a highly complex, interconnected, and dense nanoenvironment. The role of this dense nanoenvironment on molecular reactions is multifactorial as it influences accessibility, diffusion, enzyme structure, and free energy of chemical reactions^9^,^10^,^11^,^12^,^13^. Furthermore, previous work in cell-free systems and simulations has demonstrated that local density significantly regulates transcription, even non-monotonically altering expression depending on the concentration of macromolecules within the reaction volume^10^,^11^. As transcription is a probabilistic event, these probabilities will be governed in part by the physical organization within the nucleus; i.e. influenced by the physical topology of the chromatin nanoarchitecture (the spatial organization and polymeric folding of DNA, histones, and other conjugated proteins folded within the highly dense nucleus)^1^,^2^,^4^.

The physical organization of the genome is regulated at a broad range length scales, extending from the primary folding structure of DNA around histones (<10 nm) into micron scale hetero- and eu- chromatin domains within chromosomal territories. In relation to transcription, the topology of chromatin is often qualitatively characterized as compact or accessible across these length scales. For instance, studies of epigenetic repression in general qualitatively describe local compaction of chromatin near a given gene in relation to transcriptional inhibition by measuring relative changes in local accessibility to nucleases, DNA methylation (~2 nm), posttranslational modification of histones (~10 nm), nucleosome aggregation by the cohesin and polycomb complexes (50–100 nm), *et cetera*. Physically, however, all these changes converge to one common phenomenon: changes in the local density and folding of chromatin, and hence a change in nanoscale physical structure (i.e. nanoarchitecture). Indeed, the convergence of these molecular regulators on physical structure has been observed in studies of multiple chromatin remodelers (Supplemental Figure 1) and correlated with changes in accessibility^14^,^15^. Therefore, while these descriptions are intuitive for the gene under exploration, they currently do not extend into an integrated model of chromatin physical topology. For instance, consider the effect of unfolding a repressed gene to induce its expression. In dilute *ex vivo* conditions, this unfolding is not dependent on the structure of neighboring genes. However, as the radius of a gene can range from 10–100 nm and the eukaryotic nucleus is highly crowded, changes in expression for this gene will likely depends on the local folding of neighboring genes (Supplemental Information). Within this context, observations showing nanoscale transformations in nuclear topology during oncogenesis could be providing global insight into this relation across many genes. To date, however, this has not been quantitatively modeled and matched to experimental observations of expression.

In cancer, the improper regulation of transcriptional networks plays a critical role in tumor formation and metastasis. One of the common observations in tumorigenesis is the combination of the decreased activity of tumor suppressors and increased activity of oncogenic pro-growth pathways transforming healthy cells into cancerous ones^6^,^16^. While there are numerous molecular transformations that occur during oncogenesis, the physical transformation of the nucleus (and chromatin) remains the characteristic determinant of tumors independent of specific molecular drivers and a common denominator of multiple molecular neoplastic pathways. In particular, histological analysis of a wide range of tumors often identifies heterogeneity in nuclear microstructure as a determinant of tumor formation and aggressiveness. Frequently observed during tumorigenesis are variations in clumping, size, and density distribution of chromatin within transformed cells^17^. In the earliest stages of oncogenesis, previous work has shown similar changes to the physical organization of chromatin that occur at shorter, nanometer length scales demonstrating an increase in macromolecular heterogeneity^14^,^18^,^19^,^20^,^21^,^22^. Using a combination of molecular assays, transmission electron microscopy (TEM), and Partial Wave Spectroscopy (PWS) microscopy, it has been demonstrated that the nuclear nanostructure becomes more heterogeneous in the early stages of both animal models of carcinogenesis and in a wide range of human cancers, as supported by clinical studies in a few thousand patients^14^,^18^,^19^,^20^,^21^,^22^. In this context, exploration of the effect of physical structure of chromatin on the transcription of genes not only provides information about the global regulation of gene expression, but could provide mechanistic insights that links the physical and molecular transformation observed during oncogenesis.

Chromatin heterogeneity can be quantified in a number of ways. Experimental evidence has shown that physical organization within the nucleus is reasonably represented as a fractal with dimension *D*^2^,^23^,^24^,^25^,^26^. For a fractal chromatin, its fractal dimension *D* is in itself a measure of heterogeneity. Accordingly, an increase in fractal dimension has been previously observed in multiple cancers and identified as an independent prognostic marker^27^. Likewise, transformation of the fractal structure of chromatin within the nucleus has been used as an early maker for identification of tumors^28^,^29^,^30^,^31^. Taken together, these lines of evidence provide a strong empirical support to the notion that chromatin heterogeneity is a ubiquitous hallmark of pre- and cancerous cells and is associated with cancer aggressiveness as well as worse prognosis. It is of note that, in one form or another, a higher nanoscale chromatin heterogeneity has been observed in each and every types of cancer studied to date and as a common denominator of multiple molecular pathways. The implications on gene transcription, however, are poorly understood.

The fact that the chromatin nanoenvironment must play a crucial role in gene expression should not be unexpected: after all, most molecular events involved in transcription are modulated, at least to some extent, by the local density of chromatin and its global organization^32^. For instance, molecular dynamic simulations have predicted that chromatin crowding might be up- or down-regulate expression of a gene by orders of magnitude^9^. In another example, a greater surface of chromatin interface facilitates gene transcription due to, among other effects, the better access of transcription factors to DNA. This accessible surface area is a function of the local chromatin density^33^. In turn, the fractal properties of chromatin topology may have profound effects on the spatial arrangement of chromatin density. Therefore in this work, we quantitatively analyzed the effects of changes in fractal dimension *D* on the accessible surface area and the variations in focal compaction. In this model, we show that as *D* increases, both the accessible surface area and the variations of local compaction within chromatin increase. As the increase of accessible surface area and focal compaction will have competing effects on gene expression globally, we hypothesized that a competition would occur *in vitro* between activation and suppression of expression as *D* increases. Likewise, we hypothesized that increases in the variations of density would in turn produce a heterogeneity in gene expression. To test these effects, we utilized microarray analysis to measure changes in gene expression and PWS microscopy to measure the changes in chromatin heterogeneity in colonic HT-29 cells under different growth conditions. PWS microscopy quantitatively measure of the nanoscale heterogeneity through two parameters, the Disorder Strength (*L*_*d*_) and the variations of mass density (Σ), which are both proportional to *D* in chromatin. Using newly developed live cell PWS microscopy, we further show that these physical changes in chromatin structure precede the observed transformation in transcription with topological changes occurring within 30 minutes. In agreement with this model, our results show that as *D* increases a competition between gene activation and repression occurs. Additionally, the results demonstrate that increases in *D* produced an increase in transcriptional heterogeneity for critical processes such as cellular proliferation and apoptosis.

Further, to understand if these changes in genes sensitive to physical topology could have a functional significance in gene expression related to oncology, we analyzed the ontologies of genes correlated with *D*. Significantly, we show that genes highly correlated with *D* are more likely to regulate cellular metabolism than genes uncorrelated with *D* – with activation of genes regulating glucose metabolism and a suppression of mitochondrial genes maintaining oxidative metabolism, indicating a shift toward glycolytic metabolism as *D* increases. Finally, by analyzing gene expression data within the Cancer Genome Atlas (TCGA), we show that colon cancer patients with mutations in genes correlated *D* have a shorter mean survival than patients without mutations in those genes. In total, this work provides the first quantitative functional model that shows an integration between the physical structure of chromatin, transcriptional homeostasis, and colon cancer.

## Results and Discussion

In cells, there are several potential mechanisms through which changes in the physical topology of chromatin can broadly and nonspecifically regulate gene expression. For example, an overall increase in the surface area of chromatin may facilitate global gene transcription due to an improved access of transcription factors to DNA. In comparison, increasing the average mass-density (i.e. increasing the macromolecular volume fraction within the nucleus) may slow diffusion and increase the non-specific binding of transcription factors to DNA. Therefore, increasing access globally may have an associated cost that cannot be captured by qualitative models of chromatin organization. Evidence for this non-linearity between the accessible surface area and variations in focal chromatin compaction has been shown within a few tens of nanometers near the site of active transcription, suggesting that increased accessibility for some genes is paired to tightly packing neighboring genes^26^,^33^,^34^. Consequently, understanding this relation globally requires a quantitative model of chromatin physical structure.

**Figure 1:**
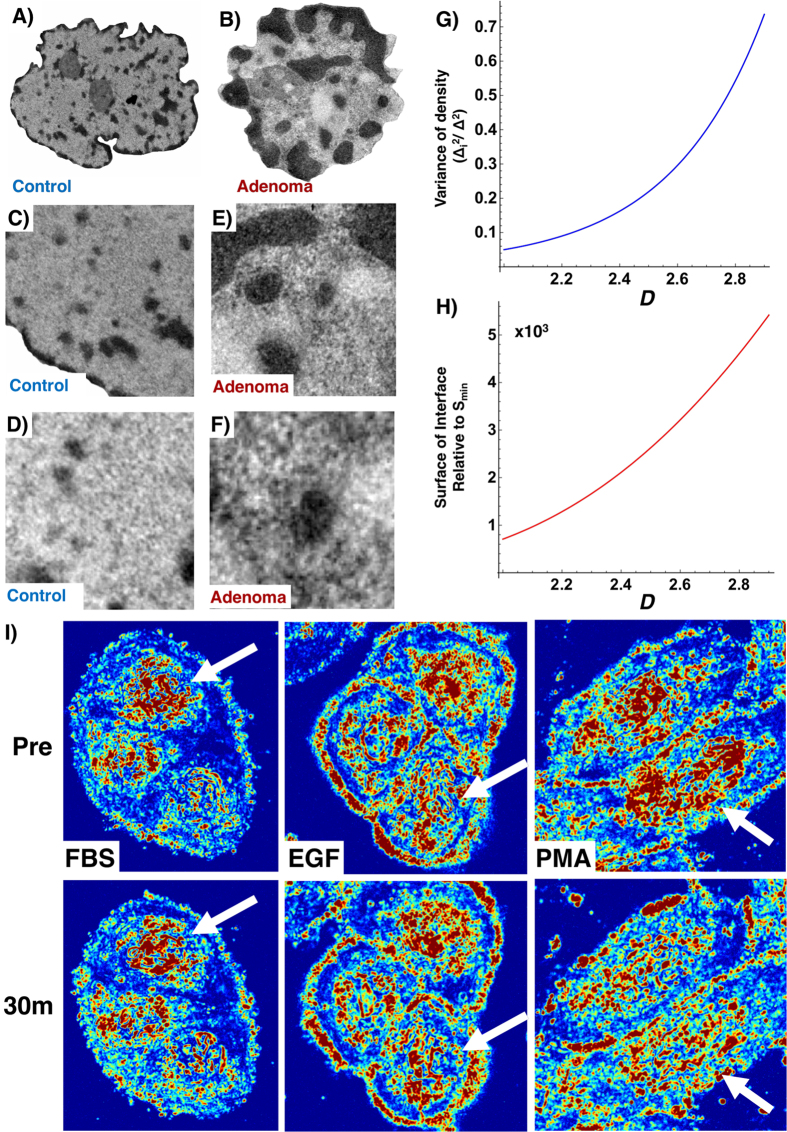
Modeling the physical structure of chromatin topology in early oncogenesis. (**A,B**) Transmission Electron Microscopy (TEM) of cell nuclei obtained from histologically normal colonic tissue in patients (**Control**) without adenoma **(A)** and from histologically normal colonic tissue in patients with an adenoma at the time presentation (**B**). (**C–F**) Representative crop from nuclei of control (**C**,**D**) and from adenoma (**E**,**F**) patients showing topological variations in nanoscopic mass density distribution qualitatively showing differences in chromatin texture. (**G**) Quantitative analysis of chromatin topology as a fractal medium shows that increases in *D* produce an exponential increase in both the surface of the interface (*S*) relative to the surface of an elementary particle (*S*_*min*_) and (**H**) increased variation in the mass density 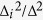. Consequently, increases in the accessible surface area within chromatin are coupled with increased focal compaction, and vice versa. To quantitatively study the transformation of physical chromatin topology within the nucleus, the mass density distribution can be modeled as a fractal medium with a characteristic dimension, *D* and *L*_*i*_ = 30 nm. Owing to the characteristic size of chromatin as a fractal globule as measured by Hi-C, the relation between M_max_:M_min_ was assumed to be at least 500,000:1. (**I)** Representative live cell PWS measurements of serum starved HT-29 cells before (Pre) and after 30 minutes after (30 m) treatment with serum (FBS), epidermal growth factor (EGF), and PMA (PMA). Arrows indicate representative nuclei.

To understand this structure-function relation in the context of human disease, we first consider the alterations that occur in the physical structure of chromatin during carcinogenesis. It is widely accepted that the physical structure of the nucleus is altered in tumor cells at the time of diagnosis. While histological identification of physical alterations in tumor cells shows evidence of micron-scale transformation in topology, the question naturally arises if this transformation extends to the earliest stages of tumor formation at the nanoscale. Previous studies using TEM and PWS have shown nanoscopic physical transformation in chromatin organization at these earliest stages even in histologically normal tissue^18^,^19^,^21^,^22^,^35^,^36^. Quantitatively, chromatin structure has been shown to behave as a fractal medium at length-scales below that of chromatin loops and the upper length scale of a chromatin globule (~250 nm). The fractal nature of chromatin folding has been observed by a variety of techniques including transmission electron microscopy (TEM)^18^, high throughput chromatin conformation capture (HiC)^2^, STORM microscopy^26^, DNA photon localization microscopy^37^, neutron scattering^23^, Partial Wave Spectroscopic microscopy^38^, and fluorescence correlation spectroscopy^12^. In carcinogenesis, previous work using TEM has shown a significant increase (p-val < 0.01) in fractal dimension in patients with pre-neoplastic colorectal adenomas in comparison to control patients^18^. Likewise, increases in *L*_*d*_ (which is directly correlated with *D*^38^,^39^, R^2^ = 0.998), have been observed in numerous types of cancer^21^,^22^,^40^,^41^,^42^,^43^,^44^. Furthermore, analysis of somatic copy number alterations in multiple tumors shows that mutational frequency correlates with a fractal organization of chromatin structure^45^,^46^. Therefore, as a qualitative illustration of the physical structure of chromatin, we begin by examining colonic cell nuclei visualized using TEM in patients with and without an adenoma present. At micron length scales, chromatin topology in patients without an adenoma show large domains of euchromatin surrounded by heterochromatin at the periphery (Fig. 1A). In comparison, nuclei obtained from histologically normal colonic tissue in patients with an adenoma show an increase in heterogeneity of structure, with variations in aggregate clusters forming throughout the nucleus immediately observable in the formation of large heterochromatin and euchromatin domains (Fig. 1B). Upon closer inspection, these qualitative differences in topology extend to the nanoscopic texture of chromatin: with sub-regions of nuclei from control patients appearing more diffuse/homogeneous (Fig. 1C and D) in comparison to sub-regions of nuclei from patients with an adenoma (Fig. 1E and F). Owing to this finding^18^ and previous studies showing that the spatial organization of chromatin is well described as a fractal at length scales that range below that formed by chromatin loops^18^,^26^,^28^,^29^,^30^,^31^, we next explored from the mathematical point of view whether these changes in fractal dimension could provide quantitative insight into the interplay between the physical structure of chromatin and transcription^2^,^12^,^26^.

Using this analysis of chromatin as a fractal medium, we quantitatively explored the dependence of nuclear fractal dimension on physical parameters of chromatin that influence transcription: (i) the surface area of chromatin, which facilitates macromolecular interactions as well as exposes DNA binding sites to transcription factors and (ii) the spatial heterogeneity of the local level of macromolecular crowding (or locally-averaged density), which could strongly influence transcription independent of binding motifs^9^,^10^,^11^. First, let us consider that for any given gene, the molecular interactions involved in its transcription occur predominantly within a specific ‘interaction volume’, *L*_*i*_^12^. Therefore, for the reasons described above, we here explore the relationship between the interaction volumes and *D* on the following parameters: the total surface area of chromatin (*S*), total variance of density throughout the nucleus (Δ^*2*^), and the variance of density averaged over the interaction volumes (Δ_***i***_^***2***^) throughout the nucleus. To derive these relations, we employed the following relationship between *S*, mass density, and *D*.

In a medium with power-law particle size distribution the cross-section of a fractal with dimension *D* is also a fractal with dimension *D*−1. Hence, the total surface area is described by the following equation relating *D* to the lower (*r*_*min*_) and upper (*r*_*max*_) limits of self-similarity:


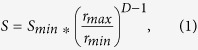


where the lower limit of self-similarity is defined by the elementary particle of the structure. Thus, the macromolecular surface area increases monotonically with the total mass (*M*) of macromolecules comprising the medium and the fractal dimension of their spatial organization. Furthermore, since the total mass is represented as:


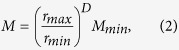


where *M*_*min*_ is the mass of the elementary particle with size *r*_*min*_, we obtain the mathematical relation between the macromolecular surface area and *D* in relation to the mass of an elementary particle:


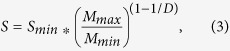


where *S*_*min*_ is the surface area of the elementary particle. As illustrated in Fig. 1G, the consequence of this relation is that increases in *D* would produces an exponential increase in the total *S* of chromatin where the elementary particle is assumed to be a single double stranded base pair and the upper limits of self-similarity is derived from chromatin conformation capture experiments showing M_max_:M_min_ is at least 500,000:1^2^.

Likewise, to calculate the relation in the variations in the local density (i.e. heterogeneous clumping) with *D,* we take into consideration that transcription occurs within a given interaction volume of size *L*_*i*_, that is much larger than the elementary particle outside of which crowding will have negligible contributions to transcriptional reactions. In relation to *D,* these variations in local density are determined by the convolution of the mass density distribution and the shape of the interaction volume, which produces the following relation (for full derivation, see Supplemental Equation 1):


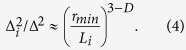


In this approximation, any crowder within the interaction volume has the same contribution independent of the distance within *Li* from the gene. Consequently, as illustrated in Fig. 1G and H, global structural reorganizations of chromatin which lead to a higher *D* would be expected to produce a twofold effect: an exponential increase in the total surface area of chromatin available for transcription processes (*S*) as well as increased variations in the local density. Hence, from the perspective of transcription, global structural reorganizations of chromatin which produce a higher *D* would be expected to produce a twofold effect that cannot be separated. On the one hand, the consequent increase in the total surface area *S* of chromatin (and hence the accessibility for genetic material to transcription factor binding) will therefore increase overall transcription. On the other hand, the overall increase in *D* is also paired with an increased degree of local compaction, which leads to repression of genes within dense clumps. As these effects are inseparably paired, the final consequence of this competition should be a general divergence (or heterogeneity) in gene expression as *D* increases.

In order to test experimentally if such a relationship exists between the physical structure of chromatin and transcription globally, we performed PWS microscopy to measure physical topology and microarray gene analysis to measure gene expression in colonic HT-29 cells grown under different conditions. In brief, PWS microscopy measures the properties of the intracellular nanoscale architecture from 20 to 200 nm by analyzing the interference spectrum of backscattered light that results from intracellular refractive index (RI) variations within each diffraction-limited resolution voxel^38^,^39^. Since RI is proportional to the local macromolecular density, it is feasible to sense and measure the nanoscale spatial arrangement of the macromolecular structures^34^,^38^,^39^. To capture this information, an interference signal between a reference wave and scattering from the RI variations within a volume defined by the spatial coherence in the transverse plane and the cell thickness longitudinally is recorded. Analysis of the back-scattered light in turn produces two structure parameters, the Disorder Strength (*L*_*d*_) and the variations of mass density (Σ), both of which measure the nanoscale heterogeneity of mass density at length scales ranging between 20–200 nm^38^. This capability for nanoscopic analysis is derived from the underlying relation between the scattering of light and organization of mass-density: even though particles smaller than the diffraction limit of light cannot be resolved, their organization can be analyzed by measuring the light they scatter. The measured variations of intensity in the back-scattered light from the cell nucleus are a result of the nanoscopic variations in macromolecular density of chromatin^38^,^39^. Extended further, the configurational arrangements within the nucleus that produce an increase in the *L*_*d*_ in fixed cells or ∑ in live cells that are due the increased variations in compaction of chromatin. *L*_*d*_ and ∑ are likewise proportional to two properties of macromolecular organization: the fractal dimension (*D*) and the standard deviation of the density (δ_n_)^47^. Taking advantage of the linear relationship between these parameters and *D* for biologically-relevant conditions (R^2^ = 0.998 for *D* between 2–3) we use PWS microscopy to measure changes in the fractal organization of chromatin (Supplemental Equation 1). Thus, observed alterations in ∑ or *L*_*d*_serve as an indication of a change in *D* and are visually represented under electron microscopy in the low *D* state by Fig. 1C and D and a high *D* state by Fig. 1E and F. Consequently, an increase in ∑ or *L*_*d*_ correlates with the computed increase in *S* and 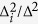 (heterogeneity of local compaction)^9^.

Owing to this relation between *D, L*_*d*_, and ∑, we hypothesized that large changes in chromatin topology as measured by Δ*L*_*d*_ or Δ∑ would capture the competition between the theorized suppression and induction of gene expression between comparative groups owing to the paired increase in *S* and 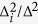. To test this relationship between the fractal topology of chromatin and gene expression, blinded PWS measurements were performed on differential growth conditions which are known to globally influence gene expression and in a knock-down model of the SWI/SNF chromatin remodeling enzyme, Arid1a. Control vector (*CV*) HT-29 (ATCC, Manassas Virginia) and HT-29 Arid-1a Kd (*A-KD*) cells were grown on glass slides under four treatment conditions: serum deprived (*SD*), serum enriched (*SE*), serum deprived supplemented with 100 nM epidermal growth factor (*EGF*), and serum deprived supplemented with 100 nM phorbol 12-myristate 13-acetate (*PMA*). These measurements produced an *L*_*d*_(in relative units to normal growth conditions for the control vector cells, *CV SE*) for each population thereby pairing these global measurements of chromatin structure with transcriptional activity by performing microarray analysis of mRNA expression using Illumina HG12-T chips. In total, fixed paired PWS microscopy and microarray analysis was performed on six groups (*CV SE, CV SD, EGF, PMA, A-KD SE, and A-KD SD*) described above with four technical replicates per group. In addition to these paired fixed population measurements, live cell PWS microscopy was performed on serum starved HT-29 colonic cells before and 30 minutes after treatment with serum, EGF, or PMA (for full details, see Methods). By tracking the same cells over short periods, causal changes in chromatin topology were measured for each perturbation condition (Fig. 1I and Supplemental Figure 2).

Analyzing data from over 21,000 probes representing 12,856 genes produced 2,445 differentially expressed genes between treatment groups (>1.5 fold change with a false discovery rate (FDR) below 5%, and adjusted p-value < 0.05%, for further details of pairwise selection of genes see Methods). As the FDR in microarray analysis can be high for individual genes and our primary aim was to test our model between the fractal topology of chromatin and the global pattern of gene transcription, we focused on general patterns of the differentially expressed genes by performing comparative analysis across all possible pairwise groups. For this comparative analysis between treatment groups, we selected as a reference point significantly over-expressed genes (p-value < 0.05 relative to the mean expression for the initial condition) and analyzed their transformation in relation to the all other states.

**Figure 2:**
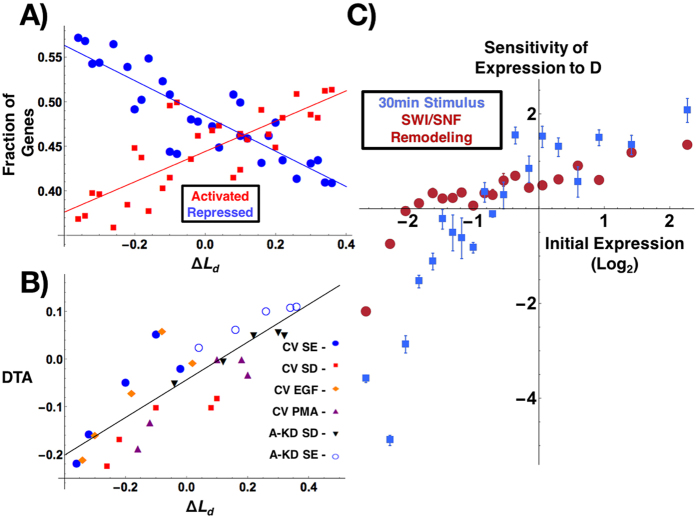
Relationship between chromatin physical topology and differential gene expression. Four growth conditions were used for control vector (CV) HT-29 cells: Serum Enriched (SE), Serum Depleted (SD), EGF stimulated (EGF), and PMA stimulated (PMA). Further, knock-down was performed on the SWI/SNF chromatin remodeling enzyme Arid1a and grown in SE (A-SE) and SD (A-SD) conditions. As ∑ and *L*_*d*_ are linear functions of *D*, PWS microscopy was used to measure nanoscopic changes in chromatin physical topology corresponding to a change in *D*. (**A**) Microarray analysis of gene expression as a function of Δ*L*_*d*_ for differentially expressed genes shows a linear correlation with fraction of upregulated genes (**Activated**, R^2^ = 0.63) and fraction of suppressed genes (**Repressed**, R^2^ = 0.75) as *D* increases. (**B**) Quantification of the summative effect on global expression was performed by calculating **Differential Transcriptional Activity** (**DTA = Activated** − **Repressed**) showing a monotonic increase in the fraction of genes overexpressed as Δ*L*_*d*_ increases independent of the comparison groups. Comparisons were made between the initial state (see legend) and all other groups. R^2^ for each comparison >0.78, and 0.70 overall. (**C)** Calculation of the sensitivity of expression for genes organized as a function their initial expression in normal growth SE conditions for the SWI/SNF conditions (CV-SD, CV-SE, A-SD, A-SE) and within 30 minutes of treatment using live cell PWS microscopy (CV-SD, CV-SE, EGF, PMA). Sensitivity is calculated as the relative rate of change in expression for a gene as a function of *D* measured through the change in *L*_*d*_ or ∑ with the errorbars representing the standard error from four experimental replicates (see Methods). A positive sensitivity indicates that as *D* increases a given gene is more likely to have an increased expression. Conversely, a negative sensitivity indicates that expression of a given gene is more likely to decrease. The magnitude of the sensitivity indicates the amplitude of the expected change as a function of *D*.

**Figure 3:**
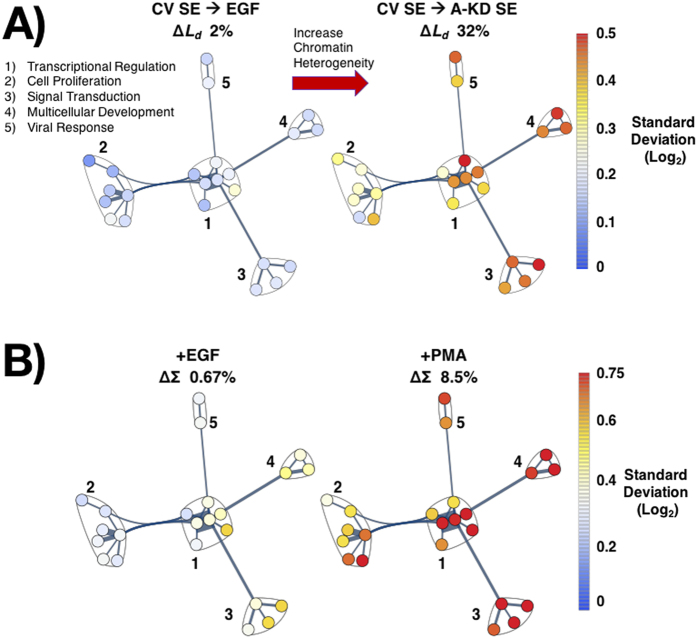
Transcriptional network heterogeneity increases with changes in the physical topology of the chromatin nanoarchitecture. (**A**,**B**) Analysis of cluster domains for 22 GO processes that contain at least 10 genes. Each point represents a GO ontological process organized by a spring-electrical distribution of ontologies. Ontologies that are highly interconnected with respect to the number of shared genes self-organize into functional domains representing: (1) Transcriptional Regulation, (2) Signal Transduction, (3) Multicellular Development, (4) Viral Response, and (5) Cellular Proliferation. Ontologies are pseudo-colored based on their intra-network heterogeneity calculated as the standard deviation of relative expression for genes, 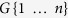, belonging to that process, ***P***, between indicated conditions: Het(***P***) = Standard Deviation 
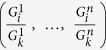
, where *k* indicates the reference condition and *i* the comparison condition. For full calculation, see Equation (6). (**A**) Intra-network heterogeneity of gene expression for the ontologies described above using *CV SE* as the reference condition in comparison to *CV EGF* (Δ*L*_*d*_ = 2%, the left graph) and in comparison to *A-KD SE* (Δ*L*_*d*_ = 32%, the right graph). Chromatin heterogeneity *L*_*d*_ was measured in fixed cells. A higher Δ*L*_*d*_ between conditions is associated with increased divergence of gene expression within any given process. (**B**) Analysis of ontological divergence as described above in relation to changes in chromatin heterogeneity in live cells measured in real-time. Transformation of intra-network expression is analyzed relative to the *CV SD* (initial state) as the reference condition to compare the transformation for two final states: + *EGF* (Δ∑ = 0.67%, the right graph) and +PMA (Δ∑ = 8.5%, the left graph). Chromatin heterogeneity Δ∑ was measured in the same live cells before and after treatment. Early transformation in chromatin topology, Δ∑, precedes observed intra-network transcriptional heterogeneity measured through microarray analysis. Critically, measurements of ∑ were taken within 30 minutes, timescales which precede the classical expectation of intra-network feedback mechanisms due to translational feedback.

Significantly, we observed that an increase in Δ*L*_*d*_ correlates with the expected increase in the fraction of overexpressed genes (R^2^ = 0.63) and decrease in the fraction of underexpressed genes (R^2^ = 0.75) independent of the treatment comparison (Fig. 2A). Furthermore, as Fig. 2B illustrates, this competitive relationship between *S* and 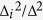 correlates with a linear relationship between Differential Transcriptional Activity (the percentage of significantly over expressed – under expressed genes (R^2^ = 0.70) and Δ*L*_*d*_. Likewise, this is most pronounced on genes with the greatest initial up-regulation and down-regulation (Fig. 2C) and in the physical transformation of chromatin in live cells within 30 minutes (Fig. 1I). Although individual genes are differentially expressed within each cohort, large groups of genes (>100 genes per group) follow a well-defined pattern that is dependent on their initial expression and the overall chromatin topology while appearing to be independent of the perturbation mechanism. Consequently, a positive sensitivity indicates that as *D* increases a given gene is more likely to have an increased expression. Conversely, a negative sensitivity indicates that expression of a given gene is more likely to decrease. The magnitude of the sensitivity indicates the amplitude of the expected change. Next, we analyzed the effect of the change in chromatin structure on the gene expression for genes belonging to the same biological process. As we observed the largest change in structure between *A-KD SE* and *CV SE* cells and the smallest change between *CV SE* and *CV EGF*, we compared the changes to the expression of their underlying networks. Critically, we found that large deviations in *D* correlate in a large degree of intra-network heterogeneity across most ontological processes, as measured by the standard deviation of relative expression (Fig. 3A). Whereas conditions with similar chromatin topologies display a similar level of expression of most of the genes within a given network, large variations in structure correlate with increased variation of expression. In live cells, transformation of chromatin heterogeneity within 30 minutes correlates with the observed level of heterogeneity of ontological networks observed at later time points by microarray analysis (Fig. 3B). Indeed, these observations are reflected by the results that stimulation with EGF (+EGF) produces minimal topological and intra-network transformation changes whereas PMA (+PMA) produces global alterations in both topology and intra-network heterogeneity. Critically, measurements of ∑ were taken from the same cells within 30 minutes, timescales which precede the classical expectation of intra-network feedback mechanisms due to translational feedback that occur over hours.

**Figure 4:**
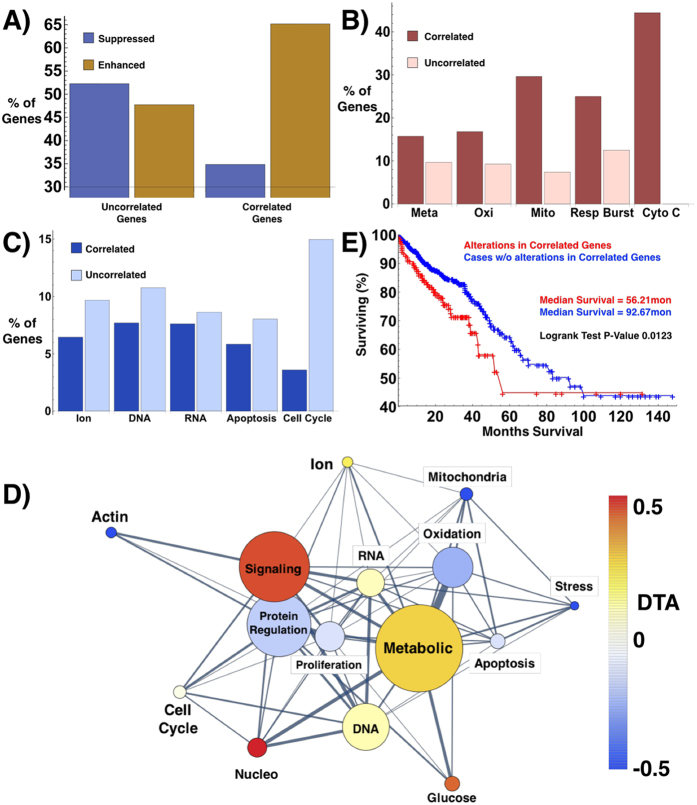
Analysis of GO process ontologies correlated with the heterogeneity (*L*_*d*_) of chromatin topology. (R^2^ > 0.80, genes in the top quantile of R^2^ values) in comparison to genes uncorrelated with *L*_*d*_ (R^2^ < 0.01, bottom quantile of R^2^) for all measured conditions (CV-SD, CV-SE, EGF, PMA, A-SD, A-SE). (**A**) Fraction of the correlated and uncorrelated genes that are either suppressed or up-regulated with the increase in *L*_*d*_. Genes correlated with *L*_*d*_ are twice as likely to be enhanced (positive slope of gene expression as a function of *L*_*d*_) as suppressed (negative slope). **(B,C**) Percentage of genes within each ontological process that are either correlated or uncorrelated with *L*_*d*_. (**B**) Genes involved in metabolism (**Meta**), including those regulating oxidation-reduction (**Oxi**), mitochondrial function (**Mito**), respiratory burst (**Resp Burst**), and Cytochrome C function (**Cyto C**) are more likely to be correlated with chromatin structure *L*_*d*_. **(C)** Genes involved in cellular homeostasis (ionic conditions, DNA and RNA binding, apoptosis, and the cell cycle) are more likely to be uncorrelated with *L*_*d*_. **(D)** Ontological processes for genes correlated with *L*_*d*_. Pseudo-color: differential transcriptional activity (DTA) for a given process (DTA = Fraction of Enhanced Genes − Fraction of Suppressed Genes). Circel size: the number of correlated genes in the process. Genes responsible for cellular metabolism (**Metabolic**), glucose metabolism (**Glucose**), nucleosome-remodeling and homeostasis (**Nucleo**), and signal transduction (**Signaling**) are more likely to be enhanced with increased *L*_*d*_. Conversely, expression of genes responsible for oxidation, stress response (**Stress**), actin remodeling (**Actin**), and protein regulation are suppressed as *L*_*d*_ increases. Genes regulating cell cycle (**Cell Cycle**), **Proliferation**, **RNA**, **DNA**, **Apoptosis**, and ionic conditions (**Ion**) are near-equally likely to be enhanced as suppressed as *L*_*d*_ increases. **(E)** Kaplan-Meier survival-curves for colorectal cancer (CRC) patients with (red) and without (bleu) mutations in genes correlated with *L*_*d*_. Thirteen *L*_*d*_-correlated genes with significant (p-value < 0.05) changes in expression within CRC patients were selected based on provisional TCGA mRNA expression. Cases with mutations in correlated genes had a median survival of ~56 months versus ~92 months for cases without mutations (p = 0.012). Cases with mutations for genes uncorrelated with *L*_*d*_ displayed no significant changes in mortality (p > 0.15).

While these results showed a strong correlation between the physical topology of chromatin and gene expression, we wanted to understand what processes, if any, were most sensitive to changes in the physical structure. To accomplish this, we characterized the GO ontologies for genes whose expression correlated with the observed changes in chromatin structure across all treatment groups. To perform this analysis while accounting for growth factor specific changes in expression, we analyzed genes highly correlated with changes in *L*_*d*_ (R^2^ > 0.8) and utilized as an internal control genes that were significantly altered but uncorrelated with *L*_*d*_ (R^2^ < 0.01). At baseline, we found that genes highly correlated with *L*_*d*_ were twice as likely to be correlated with enhancement of expression (64%) as they were with suppression (36%). In comparison, genes uncorrelated with *L*_*d*_were nearly as likely to be enhanced (48%) as suppressed (52%) (Fig. 4A). By characterizing the ontologies correlated across the multiple conditions, we explored network motifs in gene expression correlated with the global structure of chromatin. Specifically, we characterized the NCBI ontological data using inbuilt functions available in Mathematica^®^ v10 for gene functions. Of the 2445 differentially expressed genes, GO process ontologies were available for 1660 genes belonging to 1446 processes.

Interestingly, genes that are highly correlated with changes in *D* are more likely to be involved in cellular metabolism, in particular responsible for mitochondrial function, oxidative metabolism, and cytochrome C function (Fig. 4B). Conversely, genes governing metal ion homeostasis, signal transduction, DNA, RNA, cellular proliferation, apoptosis, and the cell cycle are uncorrelated with the change in *D* (Fig. 4C). To further explore functional changes, we performed an analysis of the change in expression for genes correlated with *L*_*d*_for these processes. To quantify these changes for each process, we calculated the DTA for each process. Upon analysis of differential expression of ontological processes for genes correlated with *L*_*d*_, we found that genes responsible for cellular metabolism (Metabolic), glucose metabolism (Glucose), nucleosome remodeling and nucleotide homeostasis (Nucleo), and signal transduction (Signaling) are more likely to be enhanced with an increased *L*_*d*_ (Fig. 4D). Conversely, expression of genes responsible for oxidation, stress response (Stress), actin remodeling (Actin), and protein regulation are suppressed as *L*_*d*_ increases. Additionally, genes regulating cell cycle progression (Cell Cycle), Proliferation, RNA, DNA, Apoptosis, and ionic conditions (Ion) are near-equally likely to be enhanced as suppressed as *L*_*d*_ increases. These findings, when paired with the observation of only a continuous distribution of heterogeneity within a cell population under normal growth conditions suggest a minimal dependence of chromatin heterogeneity on the stage of the cell cycle (Supplemental Figure 7). Subsequent downstream analysis of chromatin modifying genes shows a simultaneous increase and decrease in expression for genes responsible for changing the accessibility of chromatin (Supplemental Table 2). Notably, there is increased expression of core histones and the linker H1FX as *L*_*d*_ increases. Comparatively, genes involved in maintenance and folding of nucleosomes into higher-order structures show a competition between increased compaction and increased accessibility (Supplemental Table 2). For instance, both SMYD3 (which has been shown to enhance transcription of oncogenes), and SUV39H1 (which has been shown to silence transcription through heterochromatin formation) are both positively correlated with *L*_*d*_^48^,^49^,^50^. Likewise, the chromatin binding and DNA-crosslinking high-mobility group (HMG) proteins are both upregulated (HMGA1) and downregulated (HMGB1/2) as *L*_*d*_ increases^51^,^52^.

Finally, as the physical structure of chromatin is universally altered in cancer and an increased *L*_*d*_has been reported in colon as well as other types of cancer, we explored the TCGA for changes in expression of genes correlated and uncorrelated with *L*_*d*_ in patients with colorectal carcinoma (CRC). Using gene expression data from the TCGA, we selected genes significantly altered in CRC both in the correlated and uncorrelated set (Adj. p-val < 0.05). In total, 15 genes were significantly altered in the uncorrelated set whereas 13 genes were identified in the correlated cohort. Whereas patients with mutations in genes uncorrelated with *L*_*d*_showed no significant change in patient survival times in comparison to other mutations, genes correlated with *L*_*d*_had a mean survival time of ~56 months compared to 92 months for patients with other mutations (p-value 0.012, Fig. 4E). This overall difference between the cohorts may have some clinical relevance since the physical structure of chromatin is so frequently altered in oncogenesis. However, as this study is restricted to analysis of cell line models of colon cancer, additional work is required to understand if these relations extend into normal cells and other cancer models.

## Conclusions

In summary, our findings suggest that gene expression could be intimately related to the nanoscale physical organization of chromatin in a predictable way. In this context, the physical topology of chromatin may represent a molecular-pathway independent higher order chromatin ‘folding code’ which regulates the global expression of genes. In comparison with the relatively well characterized genomic and histone codes that modulate the behavior and function of individual genes, the folding code behaves similar to a “macroeconomic” modulator that acts on global patterns of expression. In that regard, the physical organization of chromatin could act as the common denominator of these patterns independent of the mode of perturbation. In our mathematical predictions as well as in our experimental findings, we observe that the nanoscale structure of chromatin could produce two divergent effects that critically regulate transcription: the accessible surface area of chromatin, *S*, and the variations of local density within the interaction volume 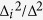. Both effects can significantly modulate transcription and cannot be uncoupled. The shift of chromatin to a high-heterogeneity state (increased fractal dimension) influences expression non-linearly; inducing a simultaneous global transcriptional activation with concurrent focal gene suppression. The effect of increased chromatin heterogeneity on the expression of gene networks is the increased variation of expression for most biological processes. Supporting the observation of increased chromatin heterogeneity correlating with gene network heterogeneity is that disruption of the SWI/SNF chromatin remodeling enzyme, Arid-1a, results in increased expression variability compared to control vector cells. Using ATP, the SWI/SNF complex proteins modulate the nanoscale organization of chromatin throughout the nucleus and are thus believed to play an important role in the transcription of genes by controlling transcription factor accessibility^53^,^54^,^55^,^56^. A possible manifestation of the transformation of chromatin structure towards a more heterogeneous configuration (Δ*L*_*d*_↑, *S*↑, Δ_i_^2^↑) could be a greater sampling of the genome. Critically, the heterogeneity of chromatin structure and genomic sampling influences critical processes such as proliferation, transcriptional regulation, signaling cascades, and cellular development. As phenotypic heterogeneity (mutational, epigenetic, and transcriptional) are determinants of tumor formation, chemoevasion, and metastasis, these findings suggest that nanoscopic physical heterogeneity of chromatin may have a significant functional contribution in these observed states^57^.

Finally, as the physical structure of chromatin is universally transformed during early tumorigenesis, we show that genes highly correlated with alterations in structural heterogeneity are more likely to regulate cellular metabolism – with activation of genes regulating glucose metabolism and a suppression of genes involved in oxidative metabolism. Interestingly, this indicates a shift toward glycolytic energy production and possibly suggests a link between structure of chromatin and the Warburg phenomena. Finally, as structure is universally altered in early carcinogenesis, we show that colon cancer patients with mutations in genes correlated with *D* have a shorter mean survival than patients without mutations in those genes. While this study does not explore the *in situ* relation between physical structure and gene expression in tissues, follow-up work addressing the integration between topological changes of chromatin in healthy, pre-malignant, and malignant cells in relation to gene expression could provide valuable insight into oncogenesis. In particular, it could expand our understanding of the factors determining transcriptional heterogeneity during tumor formation and in normal tissue. The critical implication of these results to changes is to suggest that one possible mechanism of tumor formation is heterogeneous sampling of the genetic information space due to structural heterogeneity. During early oncogenesis, repeated stress could induce inelastic transformation in the chromatin topology (i.e. increase underlying heterogeneity) that confers an advantageous sampling of the genomic landscape in addition to causing mutational transformation^57^,^58^. One level at which this occurs is to shift cellular metabolism toward a primarily glycolytic state. While this study does not directly analyze structural transformations that occurs during tumor formation and its effect on the underlying changes in gene expression through oncogenesis, it is the first demonstration that the nanoscale organization could be involved in tumor formation by altering the underlying expression of genes. Subsequently, the ubiquitously observed early transformations in the physical structure of chromatin could be more than a byproduct of tumorigenesis; it could act as one of the drivers increasing the sampling of the information space stored within chromatin. In this view, the heterogeneity of chromatin organization may mirror the heterogeneity in mutations observed in tissues during oncogenic transformation^59^,^60^. An implication is that, unlike with mutational events which would be irreversible with existing technologies, manipulation of the physical topology of chromatin could be done by physio-chemical means and utilized as a new approach to lower the risk of tumor formation by limiting the cells’ capacity for genomic sampling.

## Materials and Methods

### Cell Culture and shRNA Arid-1a KD

HT-29 Cells (ATCC, Manassas Virginia) were grown in Gibco^®^ formulated McCoys-5A Media (Life Technologies, Carlsbad California) supplemented with 10% FBS (Sigma Aldrich, St. Louis Missouri) and grown at 37 °C and 5% CO_2_. All of the cells in this study were maintained between passage 5 and 25. A lipofectamine vector was used to produce a transient HT-29 Arid-1a shRNA knockdown line (Arid-1a KD). Assessment of the knock-down was done by qRT-PCR, with imaging, flow Cytometry, and microarrays performed only on clones with over an >80% reduction in the expression of ARID-1a.

### mRNA Isolation and Microarray

To assess global changes to gene expression for each treatment group, mRNA was collected by TRIzol^®^ isolation (Life Technologies, Carlsbad California) from 10 mL petri dishes and measured by Illumina human HG12-T microarray chips. In total, six conditions were assessed with four technical replicates for each treatment condition. Quality check and the probe level processing of the Illumina microarray data were further made with R Bioconductor package, lumi by the Northwestern Genomics Core^61^. The analyzed data processing also includes a normalization procedure utilizing quantile normalization method to reduce the obscuring variation between microarrays, which might be introduced during the processes of sample preparation, manufacture, fluorescence labeling, hybridization and/or scanning^62^. Hierarchical clustering and Principal Component Analysis were performed on the normalized signal data to assess the sample relationship and variability. Probes absent in all samples were filtered out; leaving 21728 probes corresponding to 12856 genes in the downstream analysis.

### Partial Wave Spectroscopic (PWS) Microscopy

PWS measurements were performed on cells grown on uncoated glass slides at 37 C and 5% CO_2_. CV and A-KD slides were seeded at the time of passage in serum-free McCoy’s 5a medium. Before measurement, cells were treated with 100 ng/ml epidermal growth factor (EGF), or 100 ng/ml phorbol 12-myristate 13-acetate (PMA) and imaged 15 min after treatment. Measurements were performed using the optical configuration described previously^19^. In brief, light from a Xe lamp (100 W; Oriel) was focused on the sample and the back-scattered spectrum was collected by a spectrograph coupled to a CCD camera. Analysis of the fluctuations of light was performed on spectra ranging between 500–675 nm. Intensity of the backscattered light was normalized by the spectra of the incident light. After normalization, a low-pass Butterworth filter was applied to the spectra to reduce noise, which was then subtracted by a fitted second order polynomial. *L*_*D*_ was then calculated by calculating the standard deviation of the spectra divided by the correlation decay rate of the spectra as previously described^19^. Selection and analysis of cells in this study were performed in a double-blinded manner, with at least 30 cells measured per treatment group. Mean values for each group were used as a measure of the underlying chromatin nanostructure, normalized by the value for standard growth conditions (CV SE). The resulting relative *L*_*D*_ for each group was 0.9, 1.0, 0.98, 0.8, 0.64, and 0.68 for CV SD, CV SE, EGF, PMA, A SD, and A SE, respectively. Live cell PWS measurements were performed on HT-29 cells grown on 5 mm glass bottom petri dishes (Cell Vis) and serum starved for 5 hours^63^. Cells were maintained at 37 C and 5% CO_2_ for the duration of the experiment. Cells were then treated as described above with serum, EGF, or PMA for 30 minutes prior to being re-imaged. Analysis was performed on the back-scattered interference spectrum from 500–700 nm normalized by the incident light produced from the glass-media interface. A low-pass Butterworth filter was applied to reduce spectral noise and was then subtracted by a zero order polynomial to produce the heterogeneity of mass density, ∑, as calculated by the standard deviation of the intensity of the interference spectra. Transformation in chromatin structure was measured on nuclei before and after stimulation to calculate the relative change in heterogeneity after treatment over 30 minutes.

### mRNA Data Analysis

Differential gene expression between the conditions was assessed by a statistical linear model analysis using the bioconductor package *limma,* in which an empirical Bayes method is used to moderate the standard errors of the estimated log-fold changes of gene expression by the Northwestern NUSeq Core. The moderated t-statistic p-values derived from the *limma* analysis above were further adjusted for multiple testing by Benjamini and Hochberg’s method to control false discovery rate (FDR)^64^. The lists of differentially expressed genes were obtained by the FDR criteria of <5% and fold-change cutoff of >1.5. As most genes are expressed only under certain conditions, lots of genes have expression signals below the background and defined by Illumina as “absent”. Probes absent in all samples were filtered out, leaving 21728 probes corresponding to 12856 genes in the downstream analysis. Comparison groups selected to compare one degree of freedom between treatment conditions. As such, groups were compared as follows: serum starved control vector HT-29 (CV) cells vs. 10% FBS treated CV cells; serum starved CV cells vs. 100 ng/ml treated CV EGF cells; serum starved CV cells vs. 100 ng/ml PMA treated CV cells; serum starved CV cells vs. serum starved Arid-1a KD cells; serum starved Arid-1a KD cells vs. 10% FBS treated Arid-1a KD cells; 10% FBS treated CV cells and 10% FBS treated Arid-1a KD cells. From these criteria, a subselection of 2445 genes was obtained for further analysis. Calculation of sensitivity of gene expression to changes in *D* was performed by measuring the relative change in expression for each gene as a function of *L*_*d*_ or ∑. Specifically,


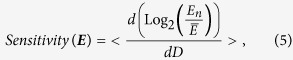


where E_n_ is the expression of any given gene and 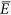 is the average expression of all genes. This produces a relation of 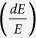 as a function of *D*. Sensitivity for each quantile is then calculated by averaging the sensitivity for all genes present within a given quantile.

### Network Ontology Analysis

Subsequent analysis of global expression changes and ontology network assessment on the 2445 differentially selected genes was performed using Mathematica^®^ v10. Ontology groups were generated using inbuilt GenomeData, matching the annotated genes with pre-defined processes and intracellular functions. Ontological information for gene processes was found for 1660 genes matching 1446 processes. Two approaches were used for analysis of genome wide expression changes: unbiased measurements of intra-network gene expression and fold-change ranked segmentation. Unbiased intra-network changes were assessed for cellular processes that contained at least 5 genes in the post-screened data. Mean-fold change, the variance of the fold-change, and Pearson correlation of the expression were measured for each process. Comparisons were performed for the following groups: A-KD and CV cells grown at 10% FBS; CV at 10% FBS and PMA treated CV cells; CV at 10% FBS and PMA treated CV cells; and serum starved A-KD and CV cells. Intranetwork heterogeneity of relative expression was measured by calculating the standard deviation of the relative expression for genes within any given ontological process. For instance, if a gene was classified as belonging to both “Chromatin Modification” and “Signal Transduction”, they were assigned to both groups and a connection between these processes was indicated. The number of connections is denoted by the thickness of the connecting line. Relative expression was calculated as the ratio of expression for a gene between the final and initial state. For any process, **P**, which contains n number of genes (**G**^**i**^), the heterogeneity (Het) of relative expression between any two conditions (**C**_**k**_ vs. **C**_**j**_) for **P** was calculated as


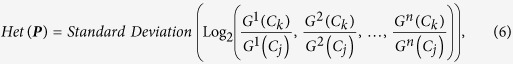


## Additional Information

**How to cite this article**: Almassalha, L. M. *et al*. The Global Relationship between Chromatin Physical Topology, Fractal Structure, and Gene Expression. *Sci. Rep.* **7**, 41061; doi: 10.1038/srep41061 (2017).

**Publisher's note:** Springer Nature remains neutral with regard to jurisdictional claims in published maps and institutional affiliations.

## Supplementary Material

Supplementary Information
