# Supplementary material for: The Global Relationship between Chromatin Physical Topology, Fractal Structure, and Gene Expression
Source: Sci Rep. 2017 Jan 24;7:41061. doi: 10.1038/srep41061 (PMC5259786; doi:10.1038/srep41061)
Supplement: Supplementary Information [file srep41061-s1.pdf]

The Global Relationship between Chromatin Physical Topology, Fractal Structure, and Gene Expression

Supplemental Information

Authors:

L. M. Almassalha[1], A. Tiwari[2], P. T. Ruhoff[3], Y. Stypula-Cyrus[1], L. Cherkezyan[1], H. Matsuda[1], M. A. Dela Cruz[2], J. E. Chandler[1], C. White[1], C. Maneval[1], H. Subramanian[1], I. Szleifer[1,4,5], H. K. Roy[2], and V. Backman[1,5]

[1]Department of Biomedical Engineering, Northwestern University, Evanston, Illinois, 60208, USA
[2]Section of Gastroenterology, Boston Medical Center/Boston University School of Medicine, Boston, Massachusetts, 02118, USA
[3]Department of Technology and Innovation, University of Southern Denmark, Campusvej 55, DK-5230 Odense M, Denmark
[4]Department of Chemistry, Northwestern University, Evanston, Illinois, 60208, USA
[5]Chemistry of Life Processes Institute, Northwestern University, Evanston, Illinois, 60208, USA
Correspondence to: v-backman@northwestern.edu

Equations 1:

Fractal dimension and the spatial heterogeneity of nuclear crowding.

To study the effect of the spatial organization of chromatin on transcriptional output, the local macromolecular interactions need to be considered. Specifically, the locally-averaged macromolecular mass-density (or the level of macromolecular crowding) is known to have a strong influence on intra-nuclear molecular interactions and transport[2-5]. Here, we study how the degree of spatial variations in the level of local macromolecular crowding depends on the fractal dimension (D) of the nucleus in relation to the lower (r_{min}) and the upper (r_{max}) limits of self-similarity.

Let us define the size of an interaction region as L_i. Then, the degree of local macromolecular crowding at a certain location within the nucleus is determined through the average mass density within the surrounding sphere of radius L_i. Since the autocorrelation of a convolution of two functions (here: mass distribution and the averaging sphere) equals convolution of autocorrelations of those two functions, we find the variance of the locally-averaged mass-

density Δ_i^2 (spatial heterogeneity of the degree of crowding) as the autocorrelation of the locally-averaged medium evaluated at the origin,

$$(1)\, \Delta_i^2 = \int B_\rho(r) \cdot A_i(r) d\vec{r}$$

where $B_\rho(r) = \Delta^2 (\frac{r_{min}}{r})^{3-D}$ is the autocorrelation of nuclear mass-density as a function of separation r, and A_i is the autocorrelation function of the volume-averaging sphere, determining the length scale where molecular interactions take place. For simplicity, we define A_i to equal $\frac{3}{4\pi}\frac{1}{L_i^3}$ at $r<r_{min}$ (to ensure $\Delta_i^2 = \Delta^2$ at small L_i) and zero elsewhere. Thus, Δ_i^2 becomes described as

$$(2)\, \Delta_i^2 = \frac{3}{4\pi}\frac{1}{L_i^3}\int_0^{L_i} \Delta^2 \left(\frac{r_{min}}{r}\right)^{3-D} d\vec{r} = \Delta^2 \left(\frac{r_{min}}{L_i}\right)^3 \left\{1 + \frac{3}{D}\left[\left(\frac{L_i}{r_{min}}\right)^D - 1\right]\right\}$$

Thus, since molecules interact at length-scales much larger than the size of an elementary particle ($L_i >> r_{min,}$), the ration between locally-averaged mass variance to total mass variance can be described in a simple form:

$$(3)\, \Delta_i^2 / \Delta^2 \approx \left(\frac{r_{min}}{L_i}\right)^{3-D}$$

In this approximation, any crowder within the interaction volume has the same contribution independent of the distance within *Li*. Consequently, the level of local macromolecular crowding is more spatially homogeneous throughout the nucleus when *D* is low, and varies strongly from one local nano-environment to another when *D* is large.

Experimental measurement of changes in D. To measure changes in *D* of chromatin for cells under different conditions, we utilized Partial Wave Spectroscopic (PWS) Microscopy. PWS microscopy captures the distribution of mass density by measuring its disorder strength L_d, which is proportional to the standard deviation of mass distribution within the sample multiplied by the characteristic length scale of its internal organization[6-8]. The disorder strength is measured via the nanoscale-sensitive spectral variance of light intensity Σ^2 registered by a wavelength-resolved white-light epi-illumination bright-field microscope with a small numerical aperture of light incidence and a large numerical aperture of collection. Σ is an approximately linear function of both the standard deviation of mass-density and the characteristic length scale of sample's

internal organization, and is also proportional to the square root of the sample thickness[25]. Thus, the disorder strength L_d is measured after normalizing Σ to eliminate its dependence on sample thickness. The general relation between any form of mass density distribution and the measured spectral variance has been previously described[9]. For fractal media such as biological cells, Σ^2 measured from a sample with fractal dimension D is described by:

$$(4)\,\Sigma^2 = RA_n 2^{\frac{D-9}{2}} * \Gamma\left(\frac{D-3}{2}\right)\left\{1-(1+(xNA)^2)^{\frac{3-D}{2}}\right\} + A_n\frac{2Rk_cL}{\sqrt{\pi}} \cdot \frac{\Gamma\left(\frac{D}{2}\right)x}{2^{\frac{3-D}{2}}(D-2)} \mathrm{x}\left\{(1+4x^2)^{1-D/2} - [1+x^2(4+NA^2)]^{1-D/2}\right\}$$

where we used the versatile Whittle-Mattern family of correlation functions to represent the material distribution within the sample (x is a unitless parameter of size with respect to the wavelength (k_cL_n, L_n denoting the upper length-scale of fractality and k_c denoting central wavenumber, A_n is the fluctuation strength of RI,NA is the numerical aperture of the microscope objective, Γ() denotes a gamma-function, R is the product of amplitude reflectance at the sample's top interface and amplitude transmission coefficients through in both directions of that interface[9]. For D ranging between 2 and 3, Σ (and, therefore L_d) is a linear function of D with an r^2 of 0.9982[3].

Derivation of Gene Size Distribution. Human genes have a broad range of lengths. For example, genes on chromosome 1 can range from as small as 43bp to as large as 1.2Mbp (**Supplemental Figure 6a**). Typically, human protein coding genes are at least 500bp in length. The transformation of this linear size into a three dimensional radius can be approximated by a number of methods and all relate to the dimensionality of structure as described above. For simplicity, we calculate the volume for genes in perfectly compacted and ideal polymer conditions. The first approximation considers DNA as an unbound polymer in solution. Here, the geometric radius of the gene can be approximated as:

$$(5)\, r = 0.15 * (BP)^k,$$

where k =1/D depends on the folding behavior of the DNA polymer, with a D ranging between 2 to 3 and 0.15 is derived from the radius of a single nucleotide. It is important to note that double stranded B-form DNA is not perfectly spherical and is typically considered to have a radius of 1nm. However, the radius of a single nucleotide in solution is ~0.15nm. We first consider a perfectly spherical nucleic acid with the radius of a single nucleotide in solution and not that of

double stranded DNA. As we describe above, D describes the folding state of polymeric DNA, where a perfectly condensed gene having D = 3 and a random loop polymer in ideal uncompressed conditions has D = 2. In the scenario where the radius is that of a single nucleotide in solution, the 3 dimensional radius of a protein coding gene can range up to ~170nm for a polymer in ideal conditions up to 16nm for a perfectly condensed polymer. Comparably, if the radius is 1nm for a single nucleotide as is the case in B-form DNA, genes would have a three dimensional radius ranging from ~20nm to ~1100nm for a polymer in ideal conditions and ~8nm to~100nm for a perfectly condensed polymer.

A second approach would be to approximate each gene coiled first around histones into nucleosomes. In this derivation, the number of nucleosomes per genes can be estimated as one nucleosome for ~200bp (150 core nucleotides and 50 linker nucleotides) with a radius of 5.5nm per nucleosome[10]. In this scenario, the geometric size of the gene is approximated by:

$(6)\, r = 5.5 * (BP/200)^k$, for genes larger than 6Kbp.

This segmentation is due to the fact that shorter genes (for simplicity assumed to be ~30 nucleosomes) cannot behave as ideal polymers whereas longer genes can be approximated as polymers with D between 2 to 3. In this case, the upper boundary for the radius of a gene is ~435nm in idealized conditions and ~100nm when the gene is perfectly condensed. Using the measurements of D from *Bancaud et. al* in live cells, where chromatin has a D of 2.5 that ranges from 2.2 to 2.6. Likewise using measured values, the radius for the longest gene on chromosome 1 would be ~180nm when D = 292nm and ~180nm for D of 2.5 (**Supplemental Figure 6b**). These considerations for single genes extend into larger territories and domains of chromatin within the nucleus. Taking into consideration that chromatin folding extends in the same manner from 500Kbp well into the range of Mbps, it is important to analytically characterize the folding of multiple genes in relation to their surface area and compaction.

Supplemental Figure 1

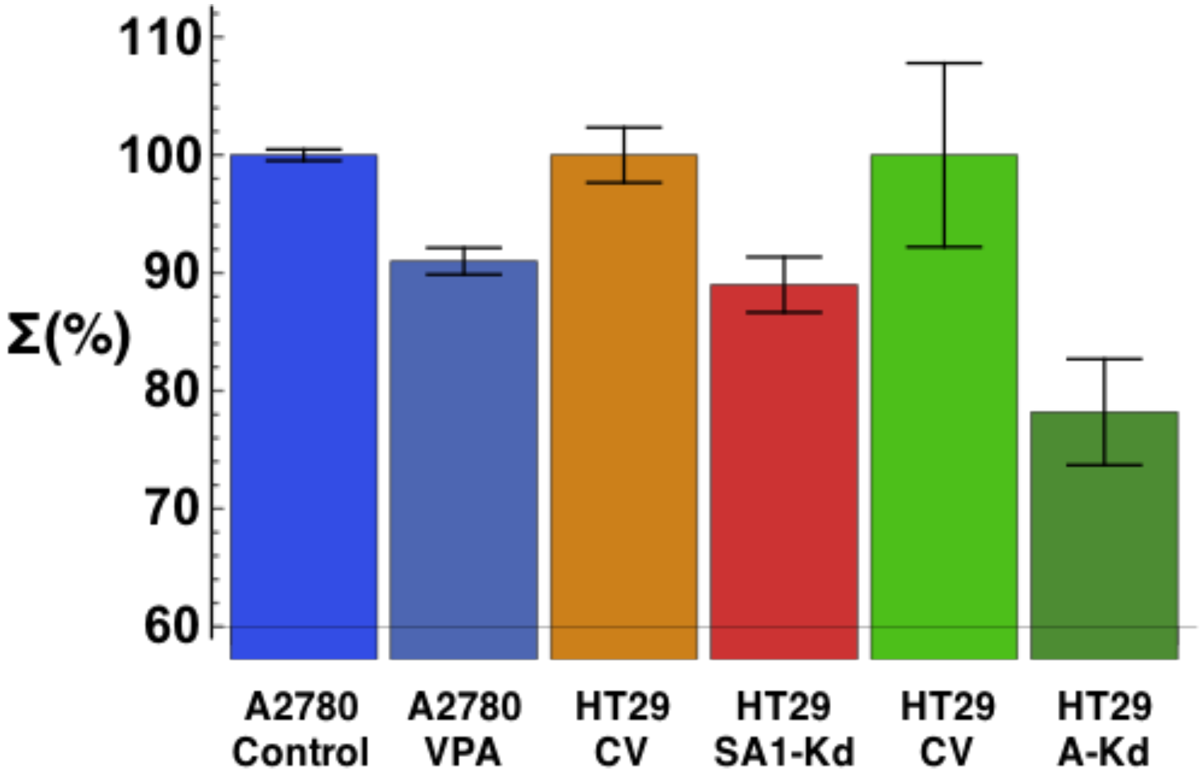

Analysis of changes in ∑ within the nucleus of cells due to known chromatin modulators. Using PWS microscopy, we have observed nanoscopic changes in topology from HDAC inhibition (VPA, p-val <0.001), shRNA suppression of cohesin SA1 (SA1-Kd, p-val = 0.002), and shRNA suppression of SWI/SNF member Arid1a (A-Kd, p-val =0.02). Key: A2780 cells treated with 100μM Valproic Acid for 30 minutes (A2780 VPA), sh-RNA knockdown of SA1 (HT29 SA1 kd), sh-RNA knockdown of Arid1a (HT29 A-Kd). All ∑ values were normalized to the associated experimental control group.

Supplemental Figure 2

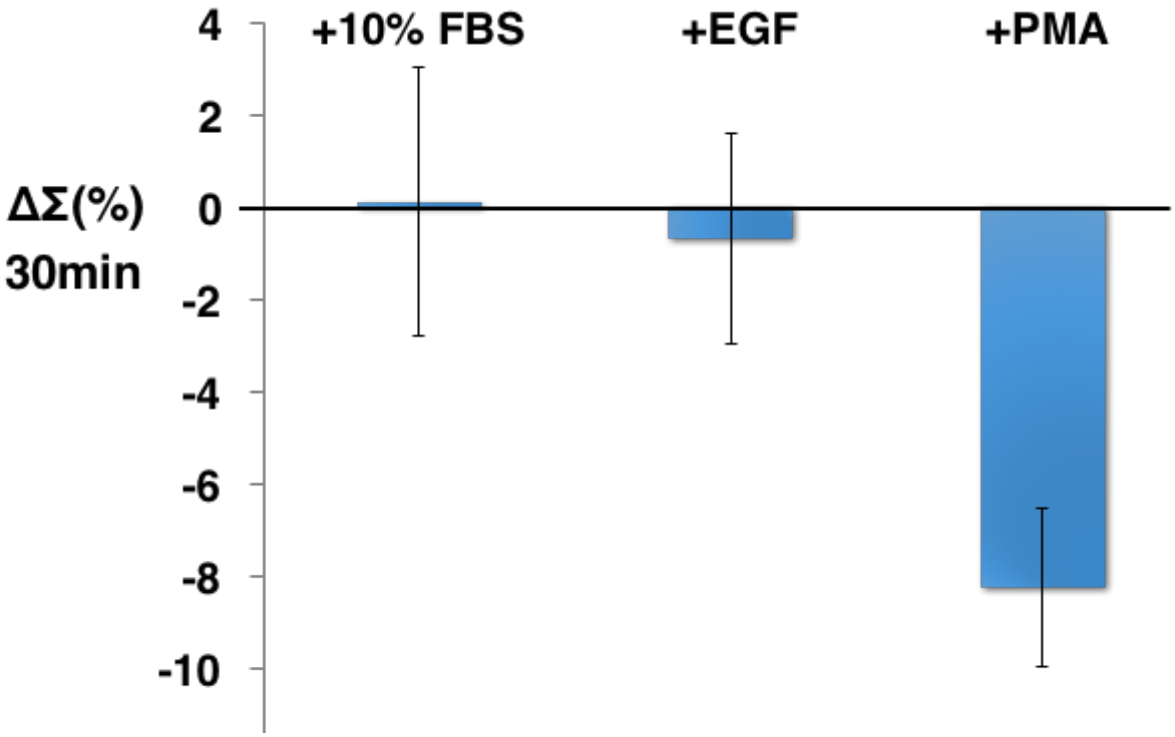

Changes in the chromatin structure in live cells after stimulation. Live cell PWS microscopy was performed on serum starved HT-29 cells before and after stimulation with serum (+10 FBS, epidermal growth factor (+EGF), and phorbol 12-myristate 13-acetate (+PMA) for 30 minutes. Measurements were taken on the same cells before and after treatment. Significantly, changes in the chromatin nanoarchitecture at the length-scale ranging from 20-200nm precede transcriptional changes at 5 hours as measured by microarray expression. Measurements were taken on over 50 cells per group (n=52, n=59, and n=67 for +FBS, +EGF, and +PMA, respectively)

Supplemental Figure 3

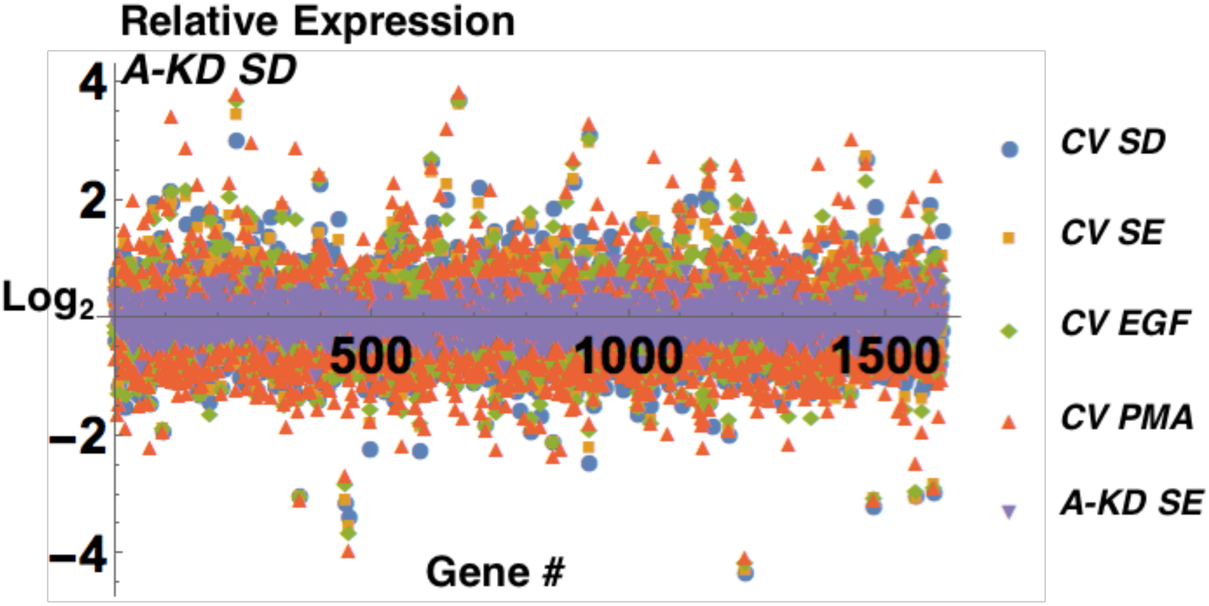

Changes in the physical organization of chromatin correlate with changes in relative expression. Relative change in gene expression between A-KD SD cells and the other treatment conditions. Small deviations in ΔL_d are manifest with small changes in gene expression. Large deviations in ΔL_d result in increasingly large changes in expression (both suppression and induction).

Supplemental Figure 4

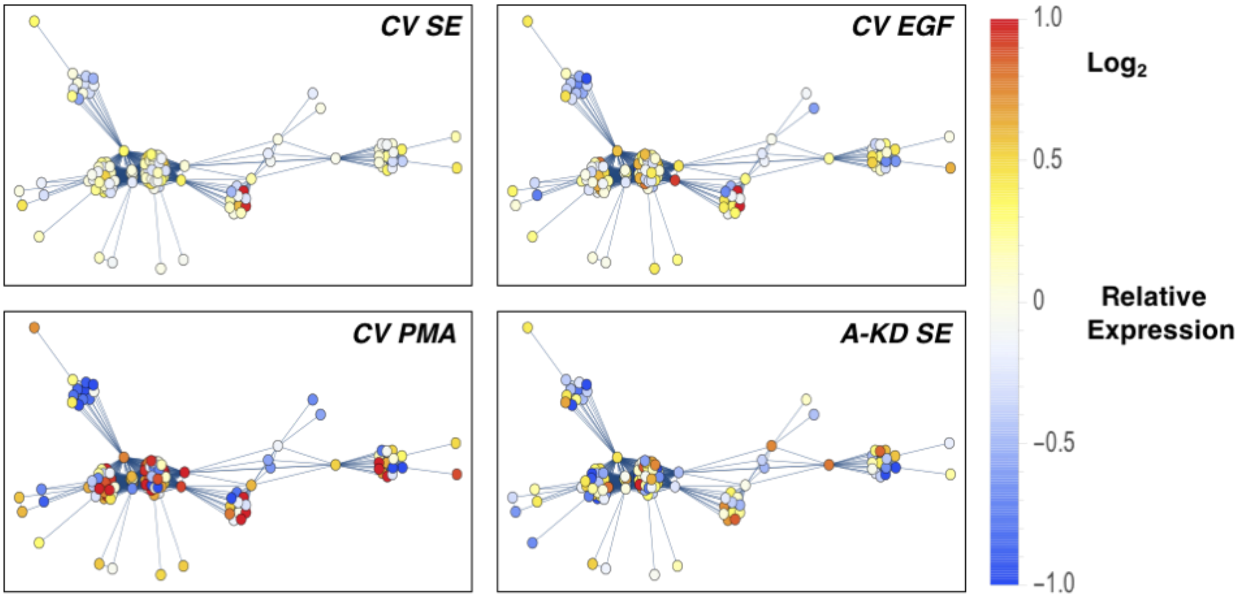

Representation of gene network topology. 100 arbitrary genes (circles) within the set are shown. Genes are grouped together by their defined process involvement (lines). Clusters of genes form based on this organization. Interestingly, changes in the structure correlate with the variation of expression of genes within given processes (See below).

Supplemental Figure 5

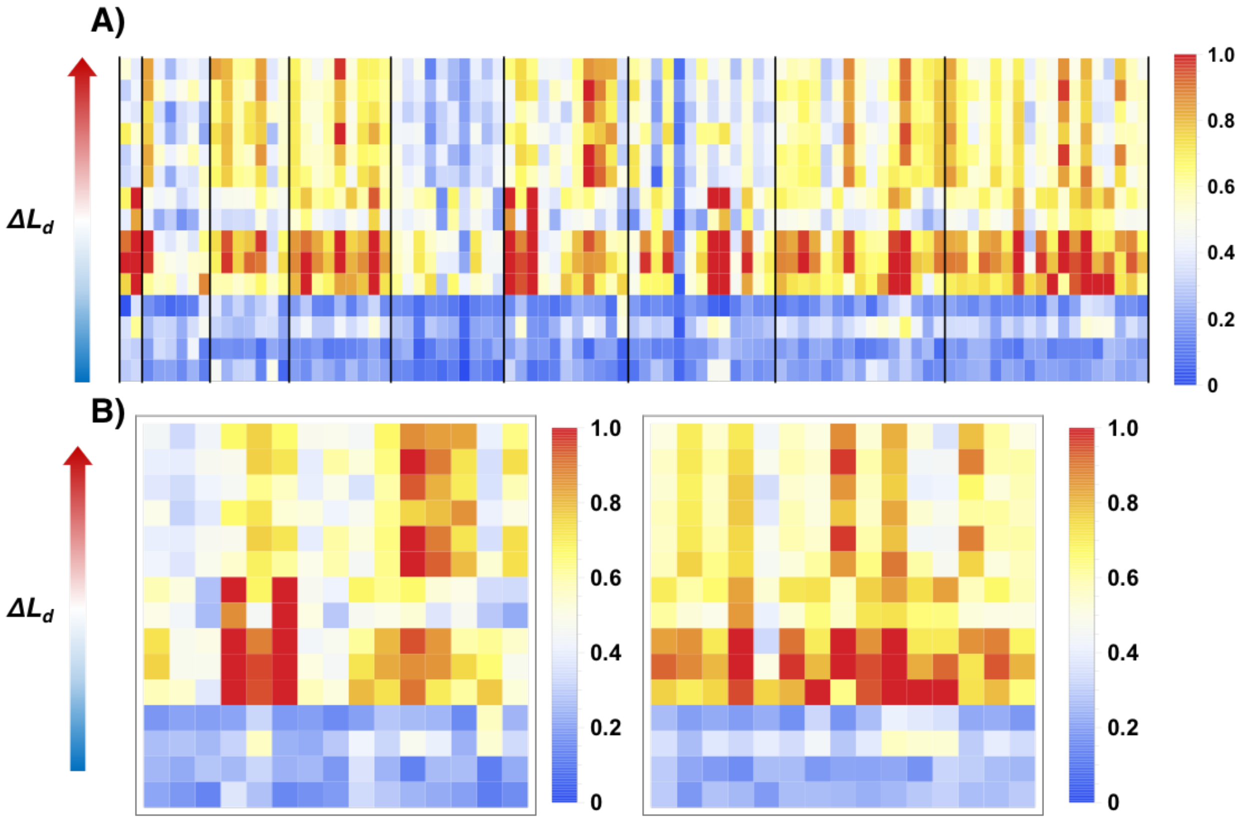

The effect of the physical structure of chromatin on heterogeneity of gene expression. (A) Full heatmap representation of the change in the standard deviation of relative expression as a function of ΔL_d for the processes identified in Figure 3. To minimize the effect of false discoveries on the result, only processes composed of at least 5 genes and have 5 genes shared with other processes. **(B)** Representative subgroups from the full set shown in (A). Interestingly, variations in gene network expression increase with ΔL_d across most processes and independent of the condition.

Supplemental Figure 6

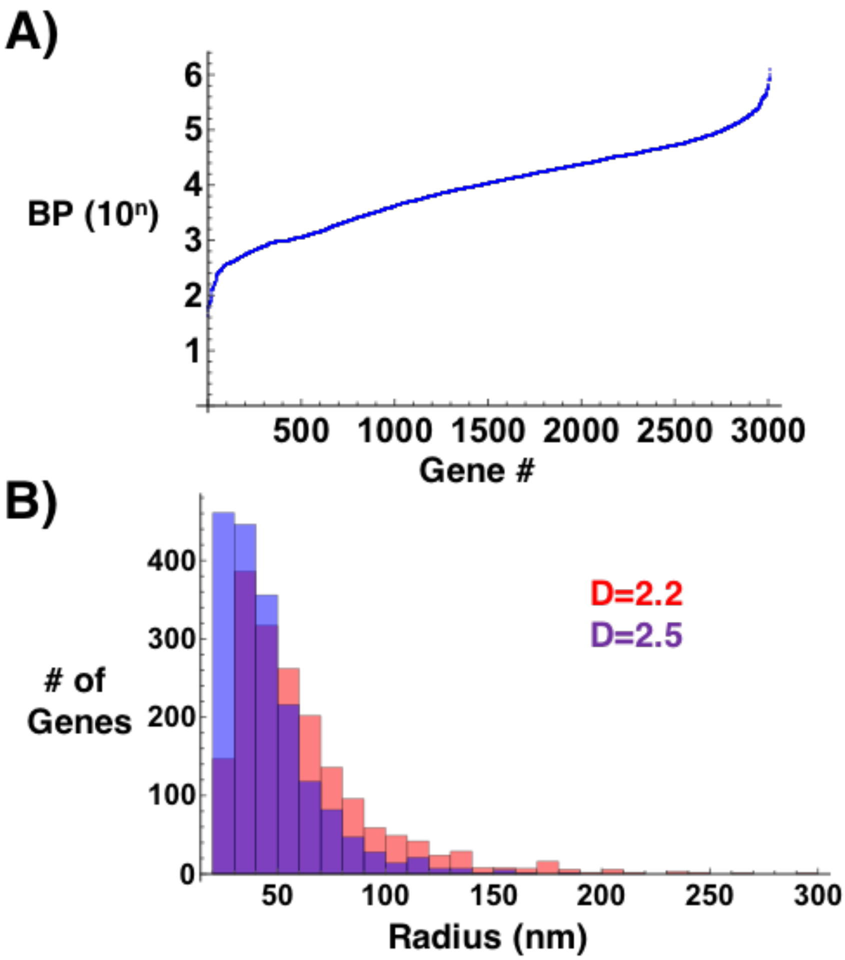

Analytical approximation of the physical size of individual genes. A) The length distribution of genes on chromosome 1 demonstrates the large size distribution of human genes. B) The theoretical radius distribution of genes in a three dimensional volume can be calculated based the folding structure of chromatin. Using previous measurements of *D* in live cells for compact and accessible regions of chromatin provides approximate sizes for genes based on the folding properties of chromatin as a polymer.

Supplemental Figure 7

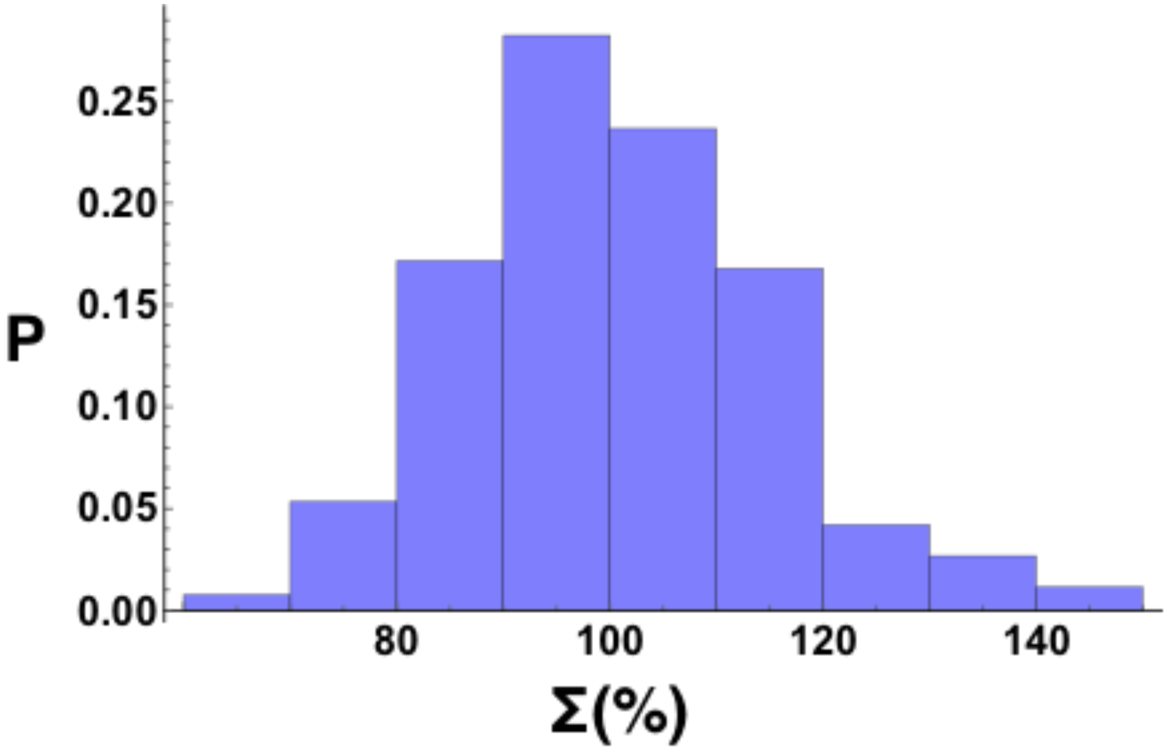

Distribution of nuclear ∑ for 262 HCT-116 cells measured from 3 independent experiments. In normal growth conditions, the cellular population shows a continuous distribution of nuclear ∑. In contrast, the observation of a bimodal distribution would suggest that stage of the cell cycle contributes significantly to chromatin organization at steady state.

Supplemental Table 1

ActinCytoskeletonOrganizationAndBiogenesis	15
AnaphasePromotingComplexDependentProteasomalUbiquitinDependentProteinCatabolicProcess	15
AnatomicalStructureMorphogenesis	12
Angiogenesis	13
AntiApoptosis	33
Apoptosis	48
BloodCoagulation	14
BrainDevelopment	12
CalciumIonTransport	12
CarbohydrateMetabolicProcess	37
CaspaseActivation	13
CellAdhesion	75
CellCellSignaling	28
CellCommunication	12
CellCycle	91
CellCycleArrest	15
CellDifferentiation	37
CellDivision	49
CellMatrixAdhesion	14
CellMotility	23
CellProliferation	56
CellSurfaceReceptorLinkedSignalTransduction	21
Chemotaxis	17
ChromatinModification	13
ChromosomeSegregation	12
Digestion	11

DNARecombination	13
DNARepair	48
DNAReplication	51
Endocytosis	18
EpidermisDevelopment	22
FattyAcidMetabolicProcess	17
GProteinCoupledReceptorProteinSignalingPathway	27
ImmuneResponse	59
InductionOfApoptosis	15
InflammatoryResponse	27
IntegrinMediatedSignalingPathway	13
InterspeciesInteractionBetweenOrganisms	50
IntracellularProteinTransport	29
IntracellularProteinTransportAcrossAMembrane	11
IntracellularSignalingCascade	36
IonTransport	48
LipidMetabolicProcess	46
MetabolicProcess	83
Mitosis	38
MRNAProcessing	11
MulticellularOrganismalDevelopment	71
NegativeRegulationOfCellCycle	31
NegativeRegulationOfCellGrowth	11
NegativeRegulationOfCellProliferation	36
NegativeRegulationOfTranscriptionFromRNAPolymeraseIIPromoter	17
NegativeRegulationOfUbiquitinProteinLigaseActivityDuringMitoticCellCycle	16

NervousSystemDevelopment	36
NuclearMRNASplicingViaSpliceosome	12
NucleobaseNucleosideNucleotideAndNucleicAcidMetabolicProcess	22
NucleosomeAssembly	15
NucleotideExcisionRepairDNAGapFilling	11
OrganMorphogenesis	14
OxidationReduction	97
PhosphoinositideMediatedSignaling	11
PositiveRegulationOfCellProliferation	21
PositiveRegulationOfIKappaBKinaseNFKappaBCascade	21
PositiveRegulationOfTranscription	11
PositiveRegulationOfTranscriptionDNADependent	13
PositiveRegulationOfTranscriptionFromRNAPolymeraseIIPromoter	21
PositiveRegulationOfUbiquitinProteinLigaseActivityDuringMitoticCellCycle	13
ProteinAminoAcidDephosphorylation	18
ProteinAminoAcidPhosphorylation	62
ProteinFolding	30
ProteinModificationProcess	26
ProteinTransport	36
Proteolysis	59
RegulationOfApoptosis	25
RegulationOfCellGrowth	21
RegulationOfCellProliferation	12
RegulationOfCyclinDependentProteinKinaseActivity	14
RegulationOfTranscription	19
RegulationOfTranscriptionDNADependent	190

RegulationOfTranscriptionFromRNAPolymeraseIIPromoter	39
ResponseToDNADamageStimulus	12
ResponseToHypoxia	15
ResponseToOxidativeStress	16
ResponseToStimulus	11
ResponseToStress	19
ResponseToVirus	31
RNAProcessing	13
RNASplicing	24
RRNAProcessing	11
SensoryPerceptionOfSound	17
SignalTransduction	156
SmallGTPaseMediatedSignalTransduction	24
SodiumIonTransport	12
Spermatogenesis	11
SteroidMetabolicProcess	12
SynapticTransmission	12
Transcription	159
TranscriptionFromRNAPolymeraseIIPromoter	26
Translation	21
TranslationalElongation	20
TransmembraneReceptorProteinTyrosineKinaseSignalingPathway	14
Transport	63
UbiquitinDependentProteinCatabolicProcess	17
VesicleMediatedTransport	22
VisualPerception	15

Processes influenced by power-law structure of chromatin organization. Representative subgroups that contain over 10 genes from the full set of 1400+ processes identified in this study. While numerous processes are influenced, changes in chromatin structure influence genes involved in cellular metabolism, ion transport, signal transduction, stress response, apoptosis, cell cycle arrest, and proliferation. Table generated from Mathematica v10.1 using inbuilt convention for text.

Supplemental Table 2

Gene	Sensitivity to Ld	RSquared	Chromatin Regulatory Function
PRMT5	-2.34174	0.639631	Methyltransferase
SMARCC2	-2.3258	0.691084	SWI/SNF Remodeling
RUVBL2	-2.02922	0.769298	PcG
HMGB2	-1.98958	0.508344	DNA Binding
MCM2	-1.88576	0.33146	Replication Loading
RBBP4	-1.81635	0.625421	HDAC
ING3	-1.76096	0.399193	HAT
CARM1	-1.71651	0.589679	HAT
HMGB1	-1.29642	0.364008	DNA Binding
MTA2	-1.2567	0.292033	HDAC
BRD8	-1.04423	0.286265	HAT
KAT2A	-0.950961	0.195047	HAT
RBBP7	-0.812011	0.116934	HDAC
CHAF1B	-0.467307	0.0350541	Replication Loading
HELLS	-0.409226	0.0199106	Heterochromatin Formation
EZH2	-0.316034	0.023925	PcG
SMARCAD1	-0.0218508	0.0000542021	Heterochromatin Maintenance
MPHOSPH8	0.0682396	0.00210668	Heterochromatin Maintenance
UTP3	0.199463	0.0153036	Gene Silencing
NAP1L1	0.271325	0.00686898	Replication Loading
EP400	0.701228	0.16009	HAT
SMARCA5	0.724528	0.121618	Heterochromatin Maintenance
SUV39H1	0.952391	0.662613	Heterochromatin Maintenance
H1FX	1.11666	0.642223	Linker Histone
HMGA1	1.12966	0.652741	DNA Coiling
SMYD3	1.1466	0.432832	Histone Methyltransferase
HIST1H2AC	1.14806	0.479869	Core Histone
H2AFJ	1.15294	0.751806	Core Histone
SMARCE1	1.36596	0.281367	SWI/SNF Remodeling
H2AFX	1.48109	0.973563	Core Histone
HIST2H2BE	1.54831	0.477479	Core Histone
HIST2H2AA3	1.55496	0.424461	Core Histone
HIST2H2AC	2.18088	0.489211	Core Histone
HIST3H2A	2.46765	0.693981	Core Histone
ARID1A	5.69283	0.814449	SWI/SNF Remodeling

Supplemental Table 2) Changes in expression for chromatin modifying enzymes as a function of changes in physical topology. While core and linker histones were positively correlated with L_d, expression patterns for other chromatin modifying enzymes show a competition between accessibility and compaction of chromatin. Key: Polycomb group proteins (PcG), Histone Deacetylase (HDAC), Histone Acetyltransferase (HAT).

References:

1 Dathe, A. & Thullner, M. The relationship between fractal properties of solid matrix and pore space in porous media. *Geoderma* **129**, 279-290 (2005).

2 Batra, J., Xu, K., Qin, S. & Zhou, H. X. Effect of macromolecular crowding on protein binding stability: modest stabilization and significant biological consequences. *Biophys J* **97**, 906-911, doi:10.1016/j.bpj.2009.05.032 (2009).

3 Bancaud, A. *et al.* Molecular crowding affects diffusion and binding of nuclear proteins in heterochromatin and reveals the fractal organization of chromatin. *EMBO J* **28**, 3785-3798, doi:10.1038/emboj.2009.340 (2009).

4 Matsuda, H., Putzel, G. G., Backman, V. & Szleifer, I. Macromolecular crowding as a regulator of gene transcription. *Biophys J* **106**, 1801-1810, doi:10.1016/j.bpj.2014.02.019 (2014).

5 Kim, J. S., Backman, V. & Szleifer, I. Crowding-induced structural alterations of random-loop chromosome model. *Phys Rev Lett* **106**, 168102, doi:10.1103/PhysRevLett.106.168102 (2011).

6 Cherkezyan, L., Subramanian, H. & Backman, V. What structural length scales can be detected by the spectral variance of a microscope image? *Opt Lett* **39**, 4290-4293, doi:10.1364/OL.39.004290 (2014).

7 Subramanian, H. *et al.* Optical methodology for detecting histologically unapparent nanoscale consequences of genetic alterations in biological cells. *Proc Natl Acad Sci U S A* **105**, 20118-20123, doi:10.1073/pnas.0804723105 (2008).

8 Subramanian, H. *et al.* Nanoscale cellular changes in field carcinogenesis detected by partial wave spectroscopy. *Cancer Res* **69**, 5357-5363, doi:10.1158/0008-5472.CAN-08-3895 (2009).

9 Cherkezyan, L. *et al.* Interferometric spectroscopy of scattered light can quantify the statistics of subdiffractional refractive-index fluctuations. *Phys Rev Lett* **111**, 033903, doi:10.1103/PhysRevLett.111.033903 (2013).
